# Effects of Various Antiepileptics Used to Alleviate Neuropathic Pain on Compound Action Potential in Frog Sciatic Nerves: Comparison with Those of Local Anesthetics

**DOI:** 10.1155/2014/540238

**Published:** 2014-02-24

**Authors:** Yuhei Uemura, Tsugumi Fujita, Sena Ohtsubo, Naomi Hirakawa, Yoshiro Sakaguchi, Eiichi Kumamoto

**Affiliations:** ^1^Department of Physiology, Saga Medical School, Saga 849-8501, Japan; ^2^Department of Anesthesiology & Critical Care Medicine, Saga Medical School, Saga 849-8501, Japan

## Abstract

Antiepileptics used for treating neuropathic pain have various actions including voltage-gated Na^+^ and Ca^2+^ channels, glutamate-receptor inhibition, and GABA_A_-receptor activation, while local anesthetics are also used to alleviate the pain. It has not been fully examined yet how nerve conduction inhibitions by local anesthetics differ in extent from those by antiepileptics. Fast-conducting compound action potentials (CAPs) were recorded from frog sciatic nerve fibers by using the air-gap method. Antiepileptics (lamotrigine and carbamazepine) concentration dependently reduced the peak amplitude of the CAP (IC_50_ = 0.44 and 0.50 mM, resp.). Carbamazepine analog oxcarbazepine exhibited an inhibition smaller than that of carbamazepine. Antiepileptic phenytoin (0.1 mM) reduced CAP amplitude by 15%. On the other hand, other antiepileptics (gabapentin, sodium valproate, and topiramate) at 10 mM had no effect on CAPs. The CAPs were inhibited by local anesthetic levobupivacaine (IC_50_ = 0.23 mM). These results indicate that there is a difference in the extent of nerve conduction inhibition among antiepileptics and that some antiepileptics inhibit nerve conduction with an efficacy similar to that of levobupivacaine or to those of other local anesthetics (lidocaine, ropivacaine, and cocaine) as reported previously. This may serve to know a contribution of nerve conduction inhibition in the antinociception by antiepileptics.

## 1. Introduction

Neuropathic pain, one of chronic pains, which occurs as a result of the damage of the PNS or CNS, is characterized by a hyperexcitability of neurons near the injured neuronal tissues [1]. This type of pain is often resistant to analgesics such as nonsteroidal anti-inflammatory drugs and opioids and thus requires other kinds of drugs including antiepileptics for antinociception [2–4]. Local anesthetics such as lidocaine and bupivacaine have been also used for the treatment of the neuropathic pain with an expectation of the inhibition of nerve action potential (AP) conduction in humans [5–7] and animals [8, 9].

Antiepileptics have various actions including voltage-gated Na^+^ and Ca^2+^ channels, glutamate-receptor inhibition, and GABA_A_-receptor activation [4, 10]. As indicated by the local anesthetics' actions, nerve conduction inhibition is important for antiepileptics to alleviate neuropathic pain. To our knowledge, however, it has not been systematically examined yet how AP inhibitions by various antiepileptics differ in extent from each other and also how the inhibitions are distinct from those of local anesthetics. We have previously reported that local anesthetics (lidocaine, ropivacaine, cocaine, procaine, and tetracaine) reduce the peak amplitude of compound AP (CAP), which is fast conducting and sensitive to a voltage-gated Na^+^-channel blocker tetrodotoxin, in the frog sciatic nerve [11–14]. In order to know the extents of nerve conduction inhibitions by antiepileptics and local anesthetics, we examined the effects of various antiepileptics and a local anesthetic levobupivacaine (which exhibits a lower risk of cardiovascular and CNS toxicity than racemic bupivacaine) [15] on CAPs recorded from the frog sciatic nerve by using the air-gap method.

## 2. Materials and Methods

This study was approved by the Animal Care and Use Committee of Saga University.

### 2.1. Frog Sciatic Nerves

The method used for obtaining frog sciatic nerve preparation has been described previously [11–14]. In brief, either sex of frogs was decapitated and then pithed; thereafter the sciatic nerve was dissected from the lumbar plexus to the knee in Ringer's solution. The isolated sciatic nerve was carefully desheathed under a binocular microscope and then loosely placed in five platinum wires, which were glued to a Lucite plate, where the two ends of the nerve were tied to the wires by using threads. The plate was put on a beaker having Ringer's solution in which the sciatic nerve was soaked. The composition of Ringer's solution used was (mM): NaCl, 115.5; KCl, 2.0; CaCl_2_, 1.8; Na_2_HPO_4_, 1.3; and NaH_2_PO_4_, 0.7 (pH = 7.0).

### 2.2. Compound Action Potential

As performed previously [11–14], the Lucite plate having platinum wires attached with the sciatic nerve was moved from the beaker containing Ringer's solution to a vacant one and then CAPs were recorded in air using a preamplifier. Here, two of the platinum wires were used to record CAPs and the other two were for stimulating the sciatic nerve at a frequency of 1 Hz with a stimulator. This procedure was quickly performed at a time interval of 2 min. The data were monitored on a storage oscilloscope while being recorded on a thermal array recorder. Stimulating the sciatic nerve produced a CAP following a stimulus artifact; the peak amplitude of the CAP was measured as a difference between baseline and CAP peak level, as done previously [11–14]. The stimulus strength used to obtain a maximal amplitude CAP was in a range of 0.4–2.7 V. A conduction velocity (CV) value was determined by using the fifth wire as an additional stimulation site and then by measuring a change in time between stimulus artifact and the peak of CAP. All experiments were carried out at room temperature.

### 2.3. Drugs

Drugs used were lamotrigine (Toronto Research Chemicals Inc., Canada), carbamazepine, phenytoin, sodium valproate (Wako Pure Chemical Industries, Ltd., Osaka, Japan), oxcarbazepine, gabapentin, topiramate, and bupivacaine hydrochloride (Tokyo Chemical Industries, Co. Ltd., Tokyo, Japan). Levobupivacaine hydrochloride was kindly gifted by Maruishi Pharmaceutical Co. Ltd. (Osaka, Japan). All of drugs (except for gabapentin, topiramate, and sodium valproate which were directly dissolved in Ringer's solution) were first dissolved in dimethyl sulfoxide (DMSO) as a stock solution and then diluted to the desired concentrations in Ringer's solution immediately before use, where the concentration of DMSO was less than 2%. Drugs at concentrations larger than 10 mM were not tested, because a change in osmotic pressure may affect CAPs. The pH of Ringer's solution containing drugs was adjusted to 7.0 with NaOH.

### 2.4. Data Analysis

Concentration-response curve for the reduction of the peak amplitude of CAP in the sciatic nerve soaked with a drug was analyzed using the following Hill equation:

(1) CAP  amplitude (%  of  control) =1001+([Drug]/IC50)nH,

where [Drug] is drug concentration, IC_50_ is the concentration of drug for half-maximal inhibition, and *n*_H_ is the Hill coefficient.

Data were indicated as mean ± SEM and statistical significance was set at *P* < 0.05 using a paired or unpaired Student's *t*-test. In all cases *n* refers to the number of sciatic nerves studied. The peak amplitude of CAP before drug application was denoted as control.

## 3. Results

Effects of drugs on the CAPs were examined in a total of 175 sciatic nerves, and the peak amplitude of the CAPs averaged to be 21.9 ± 0.5 mV (*n* = 175). When measured in some of the nerves, the CAPs had CV values of 26.4 ± 0.7 m/s (*n* = 108), values comparable to those reported previously [11–14]. DMSO at 2%, a maximal concentration used in the present study, did not affect CAPs. The peak amplitude of CAP at 20 min after soaking with DMSO (2%) was 98.8 ± 1.6% (*n* = 5) of control (19.0 ± 3.7 mV); this percentage value was not significantly different from 100% (*P* > 0.05).

### 3.1. Effects of Antiepileptics on Compound Action Potential in Frog Sciatic Nerves

**Figure 1:**
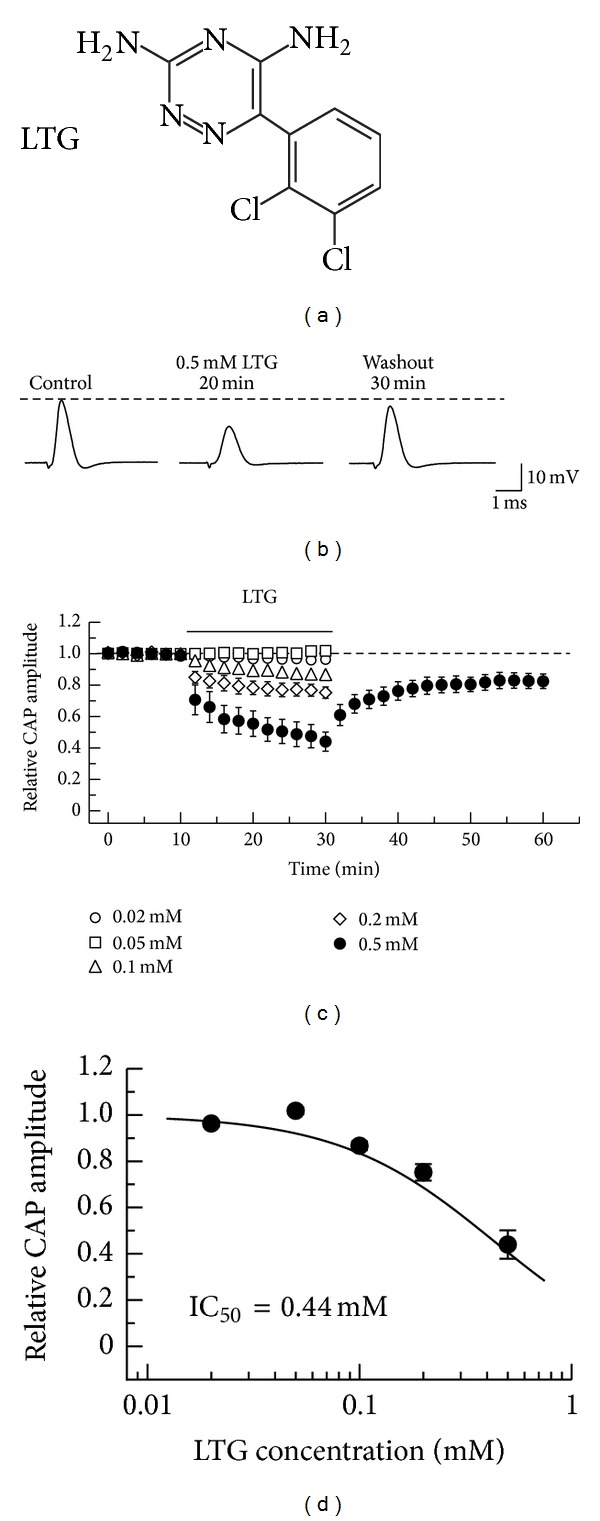
Effect of lamotrigine (LTG) on compound action potentials (CAPs) recorded from frog sciatic nerve fibers. (a) The chemical structure of LTG. (b) Recordings of CAPs before (control), at 20 min after exposure to LTG, and thereafter 30 min in the absence of LTG. (c) Average time courses of changes in CAP peak amplitude following exposure to LTG at 0.02–0.5 mM for 20 min, relative to those before the soaking (each point: *n* = 5). Relative CAP amplitude after washout of LTG is shown only for data at 0.5 mM. In this and subsequent figures, each point with vertical bars represents the mean and SEM. If the SEM of the values is smaller than the size of the symbol, the vertical bar is not shown. (d) The CAP inhibition produced by LTG is concentration dependent. The peak amplitudes of CAPs recorded from sciatic nerve fibers treated with LTG at various concentrations for 20 min, relative to control, which were plotted against LTG concentration (each point: *n* = 5). This concentration-response curve was drawn according to the Hill equation (half-maximal inhibitory concentration (IC_50_) = 0.44 mM; Hill coefficient (*n*_H_) = 1.2).

We first examined the effect of a phenyltriazine derivative (lamotrigine; 3,5-diamino-6-(2,3-dichlorophenyl)-1,2,4-triazine; Figure 1(a)), which is known to inhibit voltage-gated Na^+^ channels [16] and to relieve central poststroke pain and painful diabetic polyneuropathy [3], on CAPs in the frog sciatic nerve. As seen in Figure 1(b), soaking the sciatic nerve into lamotrigine (0.5 mM)-containing Ringer's solution reduced the peak amplitude of the CAP in a partially reversible manner.  Figure 1(c) demonstrates an average of the time courses of a change in CAP peak amplitude following soaking into lamotrigine (0.5 mM), relative to control, which is obtained from five sciatic nerves. The lamotrigine (0.5 mM)-induced reduction in CAP peak amplitude was close to a steady effect at 20 min after the soaking, where the peak amplitude of CAP was 44.0 ± 6.1% (*n* = 5; *P* < 0.05) of control (21.5 ± 2.0 mV). This percentage value was not significantly different from one (47.5 ± 7.4%; *n* = 5) at 18 min after the soaking (*P* > 0.05). At least 30 min after soaking the sciatic nerve into lamotrigine-free solution, the CAP amplitude partially recovered to control level, as shown in Figures 1(b) and 1(c). The peak amplitude of CAP at 30 min after washout of lamotrigine was 82.4 ± 4.6% (*n* = 5) of control; this percentage value was significantly smaller than 100% (*P* < 0.05).  Figure 1(c) demonstrates the time courses of changes in CAP peak amplitude with an increase in time after soaking the sciatic nerve into lamotrigine at various concentrations ranging from 0.02 mM to 0.5 mM (a maximally soluble concentration). The rate of the CAP peak amplitude reduction produced by lamotrigine was enhanced in extent with an increase in its concentration. As seen in Figure 1(c), CAP amplitude reduction after 20 min treatment increased in extent with an increase in lamotrigine concentration. The concentration-response curve for the lamotrigine-induced CAP amplitude reduction obtained from many nerve trunks (*n* = 25) is given in Figure 1(d) (IC_50_ = 0.44 mM).

**Figure 2:**
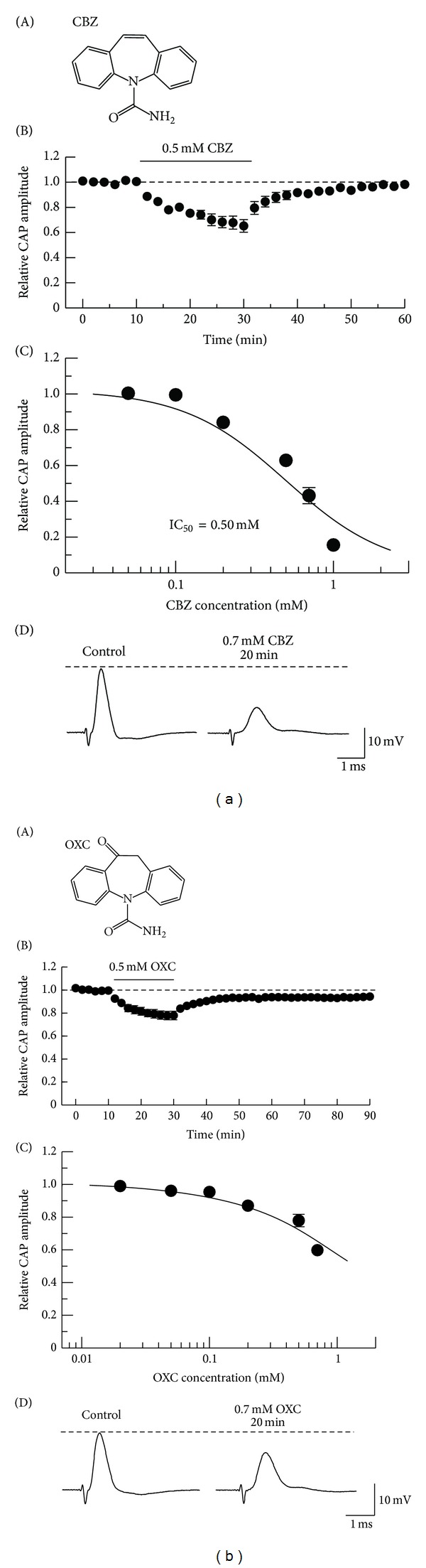
Effects of carbamazepine (CBZ) and oxcarbazepine (OXC) on CAPs recorded from frog sciatic nerve fibers. ((a)(A)), ((b)(A)) The chemical structures of CBZ ((a)(A)) and OXC ((b)(A)). ((a)(B)), ((b)(B)) Average time course of changes in CAP peak amplitude following exposure to CBZ ((a)(B)) or OXC ((b)(B)) for 20 min, relative to those before the soaking (each point: *n* = 5). ((a)(C)), ((b)(C)) The peak amplitudes of CAPs recorded from sciatic nerve fibers treated with CBZ (((a)(C)); each point: *n* = 5–9) or OXC (((b)(C)); *n* = 5) at various concentrations for 20 min, relative to control, which were plotted against its concentration. The concentration-response curve in ((a)(C)) was drawn according to the Hill equation (IC_50_ = 0.50 mM, *n*_H_ = 1.3). ((a)(D)), ((b)(D)) Recordings of CAPs before and at 20 min after exposure to CBZ ((a)(D)) or OXC ((b)(D)) at 0.7 mM. Solid line in the graph of ((b)(C)) was arbitrarily drawn.

We next examined the effect of carbamazepine (an iminostilbene derivative; 5H-dibenz[b,f]azepine-5-carboxamide; Figure 2(a)(A)), which is known to inhibit voltage-gated Na^+^ channels [17] while being different in chemical structure from lamotrigine, on frog CAPs. Carbamazepine is reported to be effective to relieve trigeminal neuralgia [18, 19]. Figure 2(a)(B) demonstrates an average of the time courses of a change in CAP peak amplitude following soaking into carbamazepine (0.5 mM), relative to control, which is obtained from five sciatic nerves. Like lamotrigine, carbamazepine exhibited an effect close to steady one of CAP amplitude reduction within 20 min after the soaking, where the peak amplitude reduced to 65.2 ± 5.0% (*P* < 0.05) of control (20.9 ± 2.6 mV; *n* = 5). This percentage value was not significantly different from one (67.8 ± 5.2%; *n* = 5) at 18 min after the soaking (*P* > 0.05). This inhibitory action was reversible, as different from that of lamotrigine. The peak amplitude of CAP at 30 min after washout of carbamazepine was 98.2 ± 1.7% (*n* = 5) of control; this percentage value was significantly not different from 100% (*P* > 0.05). Figure 2(a)(C) demonstrates the effects of carbamazepine in a wide concentration range of 0.05–1 mM on CAPs. The CAP peak amplitude reduction produced by carbamazepine was enhanced in extent with an increase in its concentration (IC_50_ = 0.50 mM).

We have previously reported that CAP inhibitions produced by tramadol and mono-*O*-demethyl tramadol [11] and also by morphine, codeine, and ethylmorphine [12] are related in extent to their chemical structures such that this magnitude is enhanced with an increase in the number of –CH_2_ in a benzene ring. We, therefore, investigated the effect of oxcarbazepine (10,11-dihydro-10-oxo-5H-dibenz[b,f]azepine-5-carboxamide; Figure 2(b)(A)) [20], where there is a keto substitution at the 10,11 position of the dibenzazepine nucleus of carbamazepine, on frog CAPs. This antiepileptic is known to be effective in relieving painful diabetic neuropathy [3] and trigeminal neuralgia [19]. Oxcarbazepine reduced the CAP peak amplitude in a partially reversible manner (Figure 2(b)(B)), an action different from that of carbamazepine while similar to that of lamotrigine. The peak amplitude of CAP at 60 min after washout of oxcarbazepine was 94.3 ± 1.5% (*n* = 5) of control (21.6 ± 0.8 mV); this percentage value was significantly smaller than 100% (*P* < 0.05). Oxcarbazepine activity was concentration dependent in a range of 0.02 mM to 0.7 mM (a maximally soluble concentration), as seen in Figure 2(b)(C). When compared at 0.7 mM, carbamazepine inhibited CAPs more effectively than oxcarbazepine (Figures 2(a)(D) and 2(b)(D)); CAP amplitude reduction by carbamazepine (43.2 ± 4.5% of control, *n* = 5) was larger than that of oxcarbazepine (59.8 ± 1.8% of control, *n* = 5; *P* < 0.05).

**Figure 3:**
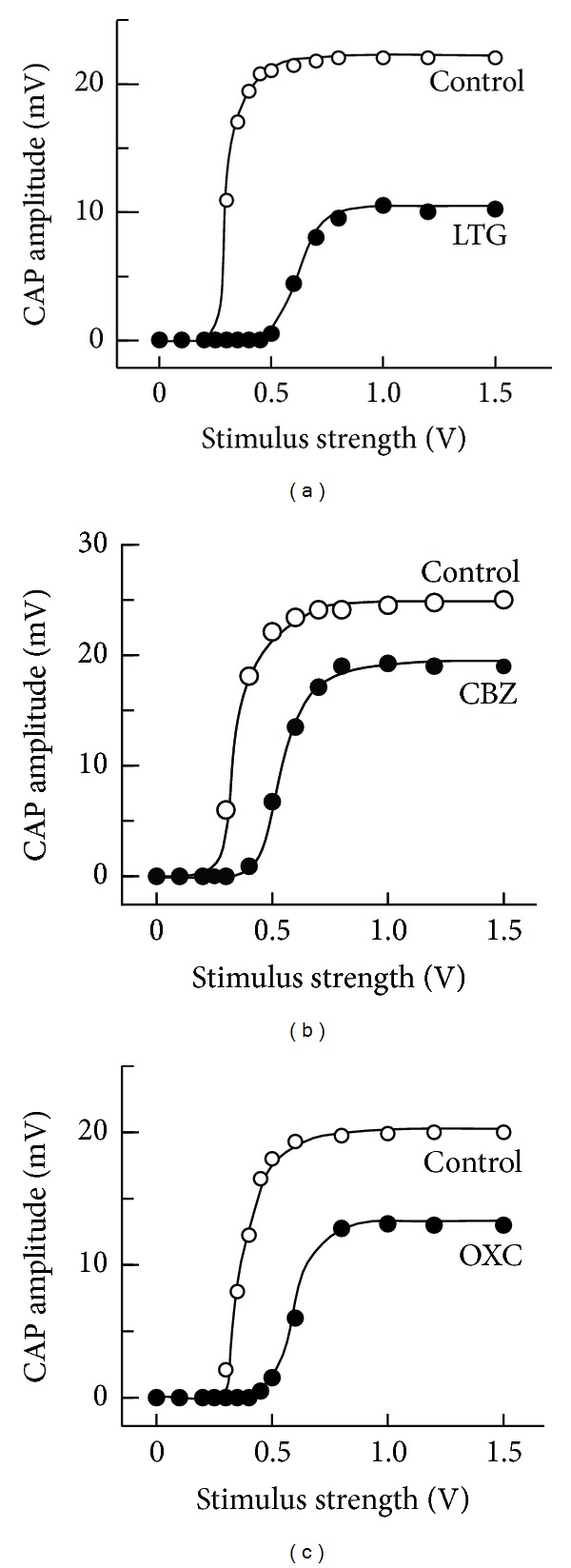
The peak amplitudes of CAPs in the absence (control) and presence of LTG (a), CBZ (b), or OXC (c); each is 0.5 mM, which are plotted against stimulus strength used to elicit the CAPs. Solid lines in the graphs of (a)–(c) were arbitrarily drawn.

Figure 3 demonstrates the effects of lamotrigine, carbamazepine, and oxcarbazepine (each 0.5 mM) on CAPs elicited at various stimulus strengths given to the sciatic nerve. Each of their inhibitory effects was seen for CAPs evoked at a maximal stimulus strength, while a threshold to elicit CAPs was increased by the antiepileptic. Each of the results was obtained in three other nerves. These results may be consistent with the observations that the antiepileptics shift the steady-state inactivation of Na^+^-channel currents to a more negative membrane potential [21–23].

**Figure 4:**
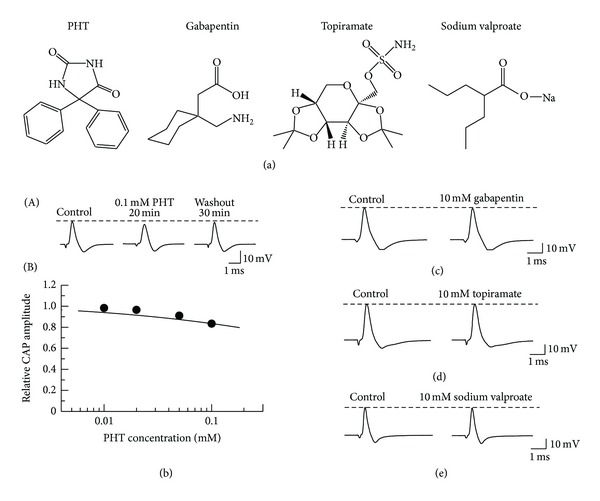
Effects of phenytoin (PHT), gabapentin, topiramate, and sodium valproate on CAPs recorded from frog sciatic nerve fibers. (a) The chemical structures of PHT, gabapentin, topiramate, and sodium valproate. (b) PHT minimally inhibits CAPs in a partially reversible manner. ((b)(A)) Recordings of CAPs before, at 20 min after exposure to PHT, and thereafter 30 min in the absence of PHT. ((b)(B)) The peak amplitudes of CAPs recorded from sciatic nerve fibers treated with PHT at various concentrations for 20 min, relative to control, which were plotted against PHT concentration (each point: *n* = 5–7). The line was arbitrarily drawn. ((c), (d), and (e)) Recordings of CAPs before and at 20 min after exposure to gabapentin (c), topiramate (d), and sodium valproate (e). They hardly affected CAPs.

A well-known antiepileptic phenytoin (hydantoin derivative; 5,5-diphenylhydantoin; Figure 4(a); which is known to inhibit voltage-gated Na^+^ channels [24] and to relieve paroxysm in trigeminal neuralgia [18]) at 0.1 mM (a maximally soluble concentration) inhibited frog CAPs in a partially reversible manner (Figure 4(b)(A)). CAP peak amplitude at 20 min after the soaking to phenytoin was 83.5 ± 1.8% (*n* = 7; *P* < 0.05) of control (16.2 ± 1.7 mV). This percentage value was also close to the steady one of CAP amplitude reduction, because this value was not significantly different from one (84.0 ± 1.9%; *n* = 7) at 18 min after the soaking (*P* > 0.05). The inhibitory action of phenytoin was concentration dependent, as seen in Figure 4(b)(B).

On the other hand, other antiepileptics, gabapentin (1-(aminomethyl)cyclohexaneacetic acid; which is related to GABA in chemical structure and reportedly relieves post-herpetic neuralgia [18]), topiramate (2,3 : 4,5-bis-*O*-(1-methylethylidene)-*β*-d-fructopyranose sulfamate, which relieves various neuropathic pains including trigeminal neuralgia and intercostal neuralgia [4]), and sodium valproate (2-propylpentanoic acid sodium salt; Figure 4(a), which provides improvement in diabetic neuropathic pain [4]), at a high concentration such as 10 mM, had no effect on CAPs (Figures 4(c), 4(d), and 4(e)). CAP amplitudes at 20 min after the soaking to gabapentin, topiramate, and sodium valproate were 97.3 ± 0.8% (*P* > 0.05; *n* = 5), 96.0 ± 1.2% (*P* > 0.05; *n* = 5), and 94.2 ± 2.4% (*P* > 0.05; *n* = 5) of control, respectively.

### 3.2. Effect of Levobupivacaine on Compound Action Potential in Frog Sciatic Nerves

**Figure 5:**
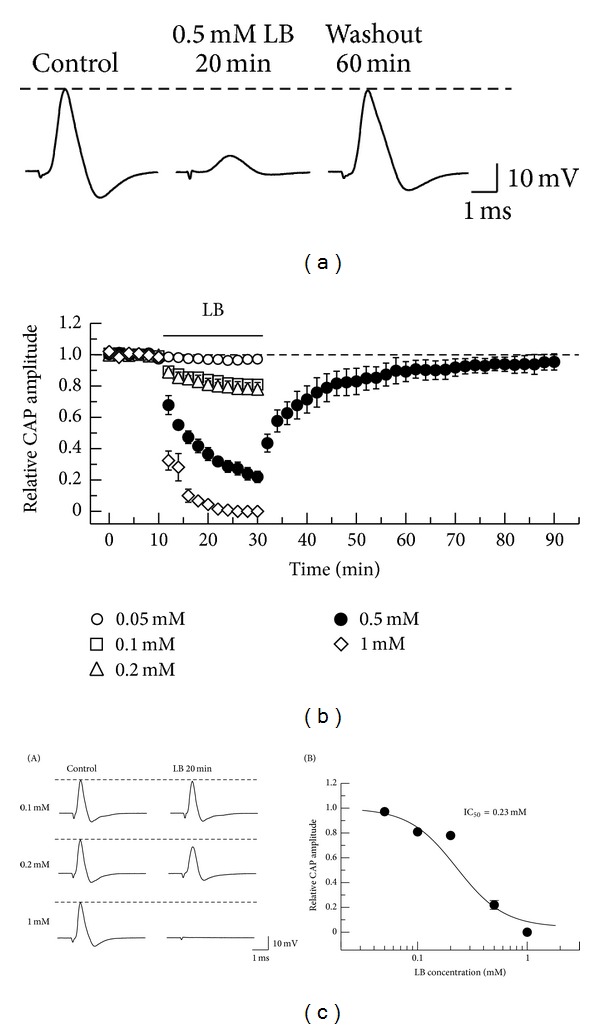
Effect of a local anesthetic levobupivacaine (LB) on CAPs recorded from frog sciatic nerve fibers. ((a), (b)) LB reduces CAP peak amplitude in a reversible manner. (a) Recordings of CAPs before, at 20 min after exposure to LB, and thereafter 60 min in the absence of LB. (b) Average time courses of changes in CAP peak amplitude following exposure to LB at 0.05–1 mM for 20 min, relative to those before the soaking (each point: *n* = 4–7). Relative CAP amplitude after washout of LB is shown only for data at 0.5 mM. (c) The CAP inhibition produced by LB is concentration dependent. ((c)(A)) Recordings of CAPs before and at 20 min after exposure to LB at 0.1, 0.2, and 1 mM. ((c)(B)) The peak amplitudes of CAPs recorded from sciatic nerve fibers treated with LB at various concentrations for 20 min, relative to control, which were plotted against LB concentration (each point: *n* = 4–7). This concentration-response curve was drawn according to the Hill equation (IC_50_ = 0.23 mM; *n*_H_ = 2.0).

In order to know whether the antiepileptics exhibit an AP inhibition comparable to those of local anesthetics, we next examined the effect of levobupivacaine on frog CAPs. Levobupivacaine at 0.5 mM reversibly reduced CAP peak amplitude, as seen in Figure 5(a).  Figure 5(b) demonstrates an average of the time courses of a change in CAP peak amplitude following soaking into levobupivacaine, relative to control. Levobupivacaine (0.5 mM) exhibited an effect close to the steady one of CAP amplitude reduction at 20 min after the soaking, where the peak amplitude reduced to 22.0 ± 3.6% (*n* = 7; *P* < 0.05) of control (22.2 ± 3.7 mV). This percentage value was not significantly different from one (24.0 ± 4.0%; *n* = 7) at 18 min after the soaking (*P* > 0.05). The peak amplitude of CAP at 60 min after washout of levobupivacaine was 95.4 ± 5.2% (*n* = 6) of control; this percentage value was significantly not different from 100% (*P* > 0.05). The extent and rate of the CAP peak amplitude reduction produced by levobupivacaine were enhanced with an increase in its concentration in a range of 0.05–1 mM (Figures 5(b) and 5(c) (A,B); IC_50_ = 0.23 mM).

Since levobupivacaine is known to be less effective in inhibiting CAPs than racemic bupivacaine [25], we next examined the effect of bupivacaine (0.5 mM) on frog CAPs. CAP peak amplitude was reduced to 23.5 ± 11.8% (*n* = 4; *P* < 0.05) of control (23.6 ± 3.1 mV) at 4 min after the soaking (not shown). This percentage value was significantly smaller than levovupivacaine's one (55.1 ± 3.2%, *n* = 7; *P* < 0.05). The treatment with bupivacaine for 20 min resulted in a complete block of CAPs.

## 4. Discussion

The present study revealed that lamotrigine and carbamazepine concentration dependently reduce the peak amplitude of the CAP with the IC_50_ values of 0.44 and 0.50 mM, respectively. This IC_50_ value for lamotrigine was similar to that (0.641 mM at −90 mV) in inhibiting TTX-sensitive human brain type IIA Na^+^ channels expressed in Chinese hamster ovary cells [16]. Consistent with our observation that lamotrigine and carbamazepine had comparable IC_50_ values, they reduced Na^+^-channel current amplitude in N4TG1 mouse neuroblastoma cells with IC_50_ values similar to each other [21]. Oxcarbazepine at 0.5 mM reduced CAP peak amplitude by 20%; this activity was much smaller than that for Na^+^-channel inhibition in differentiated NG108-15 neuronal cells (IC_50_ = 3.1 *μ*M) [23]. Smaller CAP inhibition by oxcarbazepine than carbamazepine in the present study may be consistent with the observation that oxcarbazepine was less effective than carbamazepine in inhibiting seizures induced by maximal electroshock in rats [20]. Phenytoin at 0.1 mM reduced CAP peak amplitude by only 15%. This phenytoin activity was less than those for rat cortical and human type IIA Na^+^-channel currents (60–90% amplitude reduction at −60 mV by 0.1 mM phenytoin) [16, 24]. As different from ours, phenytoin inhibited Na^+^ channels in N4TG1 mouse neuroblastoma cells with an IC_50_ value comparable to that of lamotrigine [21], and phenytoin, lamotrigine, and carbamazepine were suggested to bind to a common binding site of Na^+^ channels in rat hippocampal CA1 neurons [22]. A sensitivity of voltage-gated Na^+^ channels to phenytoin may be distinct in extent among different types of the channel. This idea is supported by the observation that there was a difference in phenytoin actions among human Na_v_1.1, Na_v_1.2, Na_v_1.3, and Na_v_1.4 *α*-subunits expressed in HEK293 cells [26]. Alternatively, there was a difference in the properties and accessibilities of Na^+^ channels between frog and rat myelinated nerves [27].

On the other hand, gabapentin and sodium valproate at 10 mM had no effect on frog CAPs. The observation that gabapentin and sodium valproate were less effective than phenytoin in inhibiting CAPs was similar to that for human type IIA Na^+^ channels [16]. Xie et al. [16] have reported that gabapentin at concentrations of less than 3 mM hardly affects the human Na^+^ channels. The antinociceptive action of gabapentin has been mainly attributed to bind to the *α*_2_ *δ*-subunit of voltage-gated Ca^2+^ channels, resulting in a decrease in Ca^2+^ entry in nerve terminals which in turn inhibits the release of neurotransmitters from there [28]. Although Zona et al. [29] reported that topiramate reduces TTX-sensitive Na^+^-channel current amplitude in rat cerebellar granule cells with an IC_50_ value of 48.9 *μ*M, frog CAPs were not affected by this drug at 10 mM. Such a result may be due to a distinction in topiramate sensitivity among different types or phosphorylation states of Na^+^ channels [30]. Each of sodium valproate and topiramate appears to exhibit antinociceptive actions through several mechanisms including an increase in GABA_A_-receptor responses [31, 32]. It is possible that the antinociceptions by topiramate and also lamotrigine are mediated by glutamate-receptor inhibition, because topiramate inhibits GluK1 (GluR5) kainate receptors in rat basolateral amygdala neurons [33] and lamotrigine inhibits AMPA receptors in rat dentate gyrus granule cells [34]. Antiepileptics having an ability to inhibit frog CAPs had a tendency to exhibit antinociception in a persistent pain model; when intraperitoneally applied in rats, lamotrigine, carbamazepine, and oxcarbazepine produced analgesic effects in the second phase of the formalin test, whereas phenytoin, topiramate, and sodium valproate did not [35, 36]. The antinociceptive effects of antiepileptics appeared to be related to their nerve conduction inhibitory actions.

**Figure 6:**
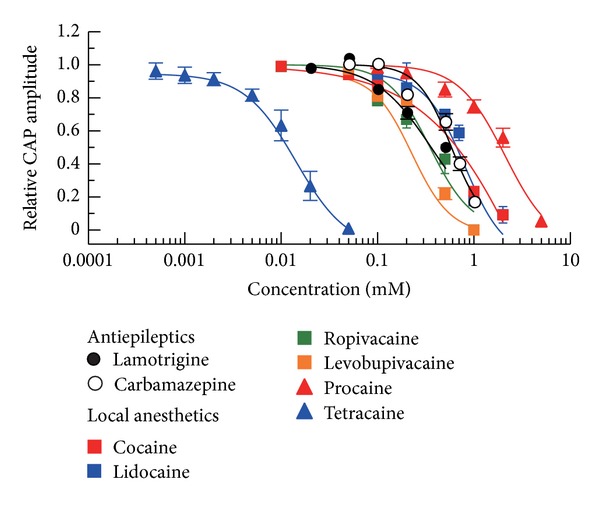
Comparison of the effects of antiepileptics (lamotrigine and carbamazepine) on frog sciatic nerve CAPs with those of local anesthetics (levobupivacaine, lidocaine, ropivacaine, procaine, cocaine, and tetracaine). The peak amplitude of CAP recorded from sciatic nerve fibers treated with each of the chemicals at various concentrations for 20 min, relative to control, which was plotted against its concentration. The data of local anesthetics except for levobupivacaine (whose data were reproduced from Figure 5(c)(B)) were taken from previous reports [11–14]. The curves for the local anesthetics were drawn according to the Hill equation, while those for lamotrigine and carbamazepine, shown by black lines, were reproduced from Figures 1(d) and 2(a)(C), respectively.

Frog sciatic nerve CAPs examined in the present study were inhibited by levobupivacaine with an IC_50_ value of 0.23 mM, a value almost comparable to that (0.22 mM) for tonic inhibition of frog CAPs by this drug [25] and also to that (0.264 mM) for tonic inhibition by this drug of voltage-gated Na^+^-channel currents recorded at −100 mV in GH-3 neuroendocrine cells [37]. Consistent with a previous report [25], this action was less than that of racemic bupivacaine. This levobupivacaine activity was almost similar to those of lamotrigine and carbamazepine.  Figure 6 illustrates a comparison of the effects of antiepileptics (lamotrigine and carbamazepine) with that of levobupivacaine and those of other local anesthetics (lidocaine, ropivacaine, procaine, cocaine, and tetracaine), as reported previously [11–14], in the frog sciatic nerve. When compared with those of other local anesthetics, IC_50_ values (0.44–0.50 mM) for lamotrigine and carbamazepine were similar to those of lidocaine, ropivacaine, and cocaine (0.74, 0.34, and 0.80 mM, resp.) while being smaller than that (2.2 mM) of procaine and larger than that (0.013 mM) of tetracaine.

## 5. Conclusion

The present study demonstrated that some of antiepileptics used for treating neuropathic pain inhibit nerve AP conduction with an efficacy similar to those of some local anesthetics, especially lidocaine clinically used for its treatment [5–7]. Nerve AP conduction inhibition by antiepileptics may be a measure of antinociception produced by the drugs in the treatment of a kind of neuropathic pain.
